# Single‐Droplet Dual‐Target Quantification of circRNA Biomarkers for Colorectal Cancer Screening

**DOI:** 10.1002/advs.202506159

**Published:** 2025-06-29

**Authors:** Jingsong Xu, Yu Liu, Junheng Zhang, Shuang Yang, Tianming Li, Haiqian Huang, Qian Liu, Hengliang Wang, Li Cao, Zhenghua An, Min Li, Hua Wang

**Affiliations:** ^1^ Department of Laboratory Medicine Renji Hospital School of Medicine Shanghai Jiao Tong University Shanghai 200127 China; ^2^ Shanghai Jiao Tong University School of Nursing Shanghai Jiao Tong University Shanghai 200025 China; ^3^ State Key Laboratory of Surface Physics Department of Physics Fudan University Shanghai 200438 China; ^4^ Department of Pathology The Fifth People's Hospital of Shanghai Fudan University Shanghai 200137 China

**Keywords:** cancer screening, circRNA, dual‐CRISPR, microfluidic chip

## Abstract

Circular RNAs (circRNAs) play a crucial role in the oncogenesis, progression, and chemoresistance of tumors. Here, two plasma circRNA biomarkers, circCK1γ3 and circWDR37 is identified and validated. Simultaneous analysis of these biomarkers has the potential to significantly improve the sensitivity of colorectal cancer (CRC) screening. Leveraging the distinct base‐cutting preferences of these two Cas proteins, it is successfully expressed and purified LwaCas13a and PsmCas13b, and developed a one‐pot Cas13a/13b‐RPA assay for the simultaneous detection of circCK1γ3 and circWDR37. To achieve precise detection of circCK1γ3 and circWDR37, the Casµchip, which integrates droplet generation and digital quantification functionalities is designed and fabricated. The Casµchip enables the simultaneous detection of circCK1γ3 and circWDR37 within 70 min. This method exhibits excellent repeatability and high specificity, with a detection limit of 100 copies/reaction. The correlation coefficients for circWDR37 and circCK1γ3 detected by Casµchip and qPCR are 0.9113 and 0.8933, respectively. Casµchip analysis is conducted on a cohort of 60 clinical samples, and the findings indicate that this methodology holds promise for the early detection of colorectal cancer. Casµchip represents the profound integration of CRISPR diagnostics and microfluidic technology, offering a novel paradigm characterized by rapid, precise, and user‐friendly features for molecular diagnosis.

## Graphical abstract

Circular RNAs are crucial in tumorigenesis. This study identifies circCK1γ3 and circWDR37 as dual plasma biomarkers for colorectal cancer (CRC) screening. It is developed Casµchip, a droplet microfluidic chip using Cas13a/b technology to quantify these biomarkers. The system integrates two Cas proteins and dual fluorescent probes for simultaneous detection of circWDR37 and circCK1γ3, establishing Casµchip as an advanced CRC screening tool.

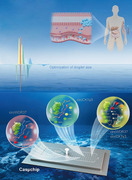

## 1 Introduction

Colorectal cancer (CRC) has the third highest incidence and second highest mortality rate of all malignancies, placing a significant burden on healthcare systems worldwide.^[^ ^1^ ^]^ Despite the high mortality rate associated with advanced‐stage CRC, the disease is also curable if detected at an early stage.^[^ ^2^ ^]^ At present, the most reliable method for screening for CRC is colonoscopy, which has been demonstrated to reduce CRC mortality statistically significantly.^[^ ^3^ ^]^ However, colonoscopy, as an invasive diagnostic and therapeutic method, has certain complexity and potential risks, which to some extent affects the compliance of some patients with the examination^[^ ^4^ ^]^ and the effectiveness of a screening strategy is contingent not only upon the efficacy of the screening test itself but also on patient adherence.^[^ ^5^ ^]^ Consequently, a variety of fecal and blood‐based screening have been developed, including carcinoembryonic antigen (CEA), carbohydrate antigen 199 (CA199), guaiac‐based fecal occult blood tests (gFOBTs), fecal immunochemical tests (FITs), multi‐target stool DNA (mt‐sDNA) tests, and methylated septin‐9 gene assays.^[^ ^6^ ^]^ Nevertheless, the aforementioned screening methodologies lack sufficient sensitivity as well as specificity for detecting early‐stage, asymptomatic CRC patients, leading to a scenario where some patients are already in advanced stages upon diagnosis.^[^ ^7^ ^]^ Hence, it is imperative to identify novel molecular markers for CRC and develop rapid, universal, and precise detection methods. This will provide a robust diagnostic foundation for clinical practice, thereby reducing the incidence and mortality rates associated with CRC.

Circular RNAs (circRNAs) represent a class of non‐coding RNAs that have been identified in the body fluids of cancer patients and hold potential as biomarkers for cancer.^[^ ^8^ ^]^ We have previously validated that plasma circWDR37 can serve as a molecular marker for CRC screening and prognostic assessment, and developed a precise detection platform named µDCR.^[^ ^9^ ^]^ However, the diagnostic sensitivity of circWDR37 as a single marker for CRC requires further enhancement. Furthermore, the µDCR technique is constrained by its capability to detect only one circular RNA (circRNA) at a time. Additionally, as droplet generation and detection are conducted on separate chips, this limitation restricts the broader application of this technology. Furthermore, a single circRNA may not be adequate for clinical application. The combined detection of multiple circRNAs, or even the integration of different types of biomarkers such as CEA and CA199, can significantly enhance the sensitivity and specificity of blood tests. This approach facilitates the development of novel and more precise screening and diagnostic methods for CRC.^[^ ^10^ ^]^

In this study, we screened and validated another circCK1γ3 that is differentially expressed in CRC. The combination of circCK1γ3 and circWDR37 improves the accuracy of CRC screening. The subsequent challenge we need to address is the development of a method for the simultaneous and accurate detection of two circRNA targets. Clustered regularly interspaced short palindromic repeat sequences‐based (CRISPR‐based) detection is ideal for multiplexing due to the distinct cleavage preferences of different Cas enzymes, particularly Cas13, which can be programmed for sequence‐specific detection using crRNA.^[^ ^11^ ^]^ Gootenberg et al. developed a one‐pot reaction that combines multiplexed reverse transcription‐recombinase polymerase amplification (RT‐RPA) and multiplexed Cas13 detection in the same tube and demonstrated a simple visualization setup for multiplexed gene detection.^[^ ^11^ ^]^ However, this methodological approach is incapable of achieving absolute quantification and simultaneous detection of multiple targets.

**Figure 1:**
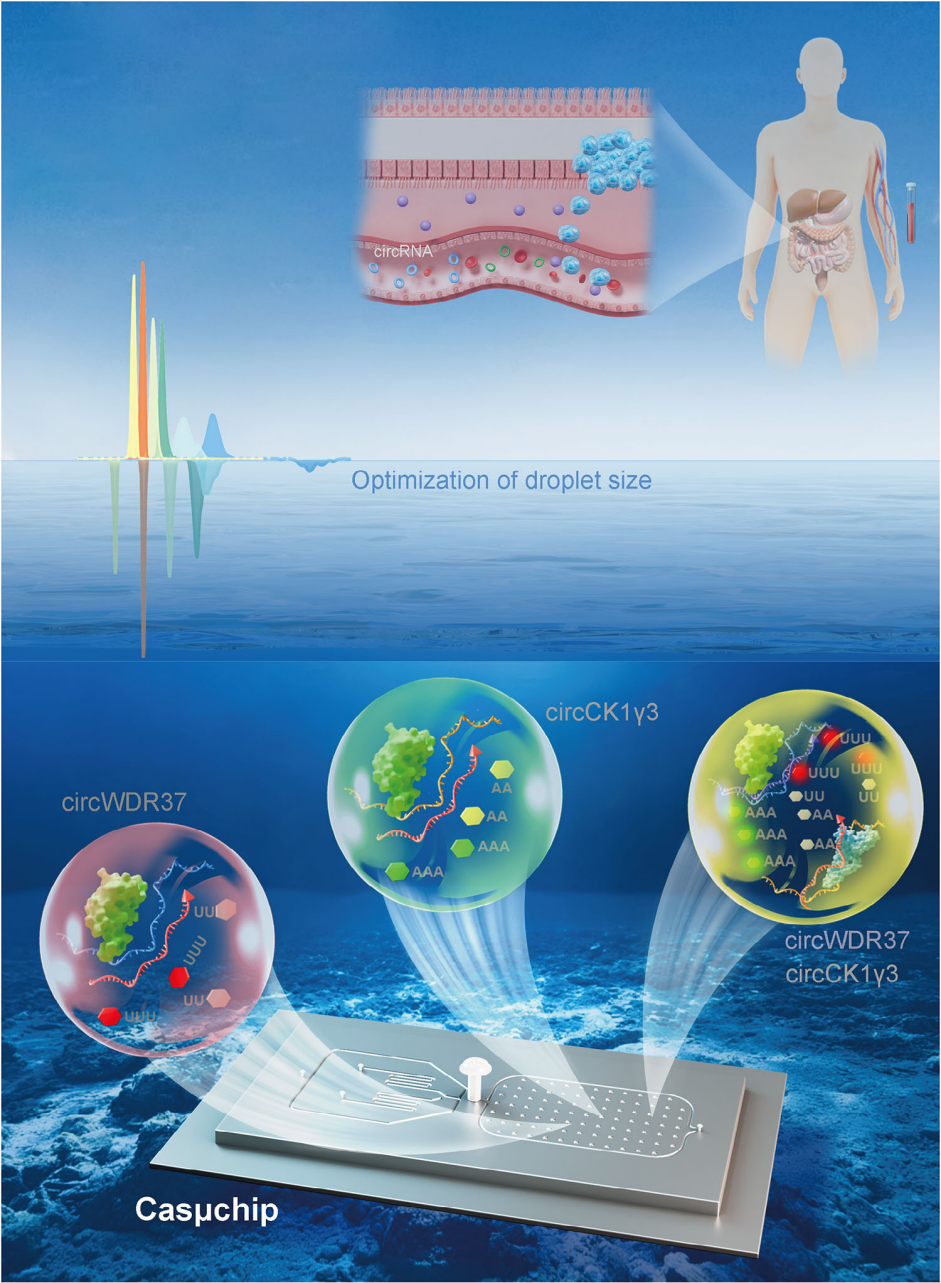
Schematic illustration of the Casµchip platform for the detection of circRNAs as biomarkers for CRC. During CRC cell proliferation, tumor microenvironment will influence the amount of circWDR37 and circCK1γ3 in bloodstream. The Casµchip enables precise quantification of these circRNAs through droplet generation and detection, ensuring uniform droplet sizes. The system integrates Cas13a and Cas13b with dual fluorescent probes allowing simultaneous identification of circWDR37 and circCK1γ3.

To address the limitations of the current CRISPR detection, we develop a droplet microfluidic chip, termed Casµchip (Cas13a/b‐based droplet microchip), for quantification of dual‐target circRNA biomarkers within 70 min. As shown in **Figure** **1**, during CRC cell proliferation, tumor microenvironment will influence the amount of circWDR37 and circCK1γ3 in bloodstream. The rapid nucleic acid extraction method can extract RNA from blood in less than 30 min. The Casµchip was engineered to enable precise quantification of target circRNAs in 40 min. Droplet generation and detection are executed on the Casµchip, ensuring uniformity in droplet quantities through precise control of droplet dimensions. The integrated detection system incorporates two Cas proteins (Cas13a and Cas13b) paired with dual fluorescent probes (CY5‐UUUUU‐BHQ2 and FAM‐AAAAA‐BHQ1), facilitating concurrent identification of circWDR37 and circCK1γ3. This innovation establishes the Casµchip as an advanced technological framework for screening of CRC.

## 2 Results

### 2.1 Identification of circCK1γ3 in CRC

**Figure 2:**
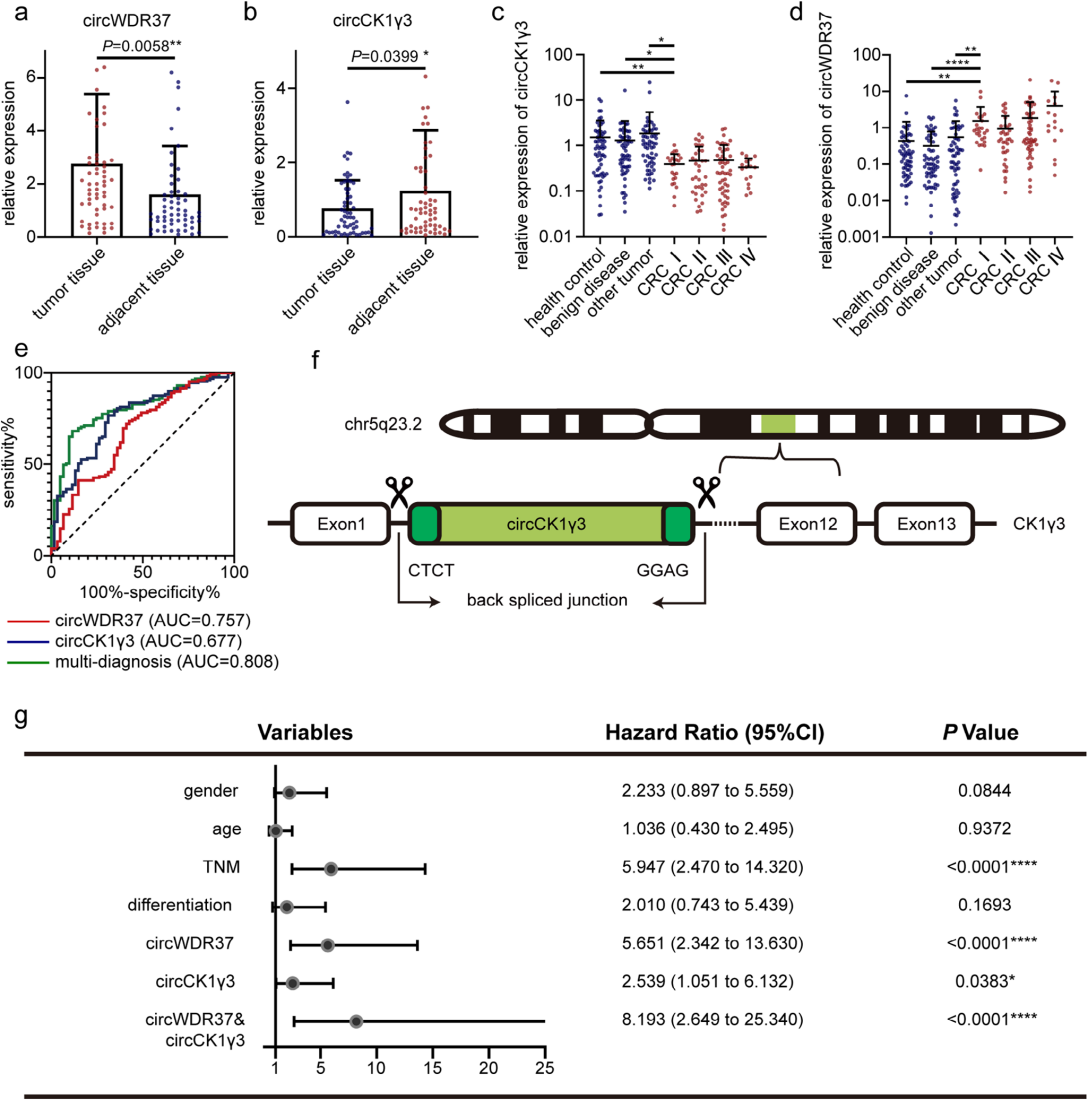
circCK1γ3 and circWDR37 are associated with CRC. a) Relative expression level of circWDR37 in tumor tissue and adjacent tissue. b) Relative expression level of circCK1γ3 in tumor tissue and adjacent tissue. Each point represents a sample and the error bar represents the mean and standard deviation. c,d) Relative expression level of circCK1γ3 and circWDR37 in plasma. CRC I represents early stage of CRC in the TNM staging system. e) ROC curve of circWDR37, circCK1γ3 and multi‐diagnosis. f) Schematic illustration of the genomic location and back splicing of circCK1γ3. g) Forest plot of hazard ratios for health variables in logistic regression analyses. Significant test was analyzed using Log‐rank (Mantel‐Cox) test.

In our previous research, we employed circRNA microarrays to identify circRNAs that exhibited significant differential expression in CRC.^[^ ^9^ ^]^ However, previous research was constrained to a subset of circRNAs that exhibited high expression levels in CRC. For instance, circWDR37 demonstrated a substantial increase in colorectal cancer tissue (**Figure** **2**a). In this study, we concentrated on circRNAs that exhibited marked under expression in CRC. The back‐splicing sites of 7 identified circRNAs were validated through polymerase chain reaction (PCR) and Sanger sequencing. To further substantiate their downregulation in CRC, we measured their expression in 60 pairs of CRC tissues. The expression level of circCK1γ3 was found to be markedly reduced in CRC tissues (Figure 2b). Nevertheless, no significant differences were observed in the remaining circRNAs between adjacent tissue and tumor tissue (Figure S1, Supporting Information). Subsequently, we sought to ascertain the presence of circCK1γ3 in the circBase and circBank database. The genomic structure of circCK1γ3 is derived from the protein‐coding locus CK1γ3, which is located at 5q23.2. It is generated through the process of back splicing, whereby the 2nd to 11th exons of CK1γ3 are joined together (Figure 2f).

As a biomarker, circCK1γ3 expressed differentially not only in CRC tissue but also in plasma which can be sampled more easily. To highlight the value of circCK1γ3 in the early screening of CRC, all CRC patients were classified into stage I to IV according to the TNM stage. Compared to healthy controls, benign diseases, and other tumors, plasma circCK1γ3 expression levels were significantly decreased in CRC I (*P* = 0.0074, *P* = 0.0414, and *P* = 0.0463, respectively) (Figure 2c). This indicates that the biomarker exhibits disease specificity. Similarly, In comparison with healthy controls, benign diseases and other tumors, plasma circCK1γ3 expression levels exhibited a statistically significant increase in CRC I (*P* = 0.0023, *P* < 0.0001, and *P* = 0.0051, respectively) (Figure 2d).^[^ ^9^ ^]^ The Receiver Operating Characteristic (ROC) analysis showed that circCK1γ3 exhibited an area under the curve of 0.677, whereas circWDR37 was 0.757 when used as the sole screening indicator for CRC (Figure 2e). Combining circCK1γ3 with circWDR37 for CRC diagnosis resulted in an AUC of 0.808, which is superior to traditional tumor marker CEA and CA199 (AUC = 0.5683 & 0.7082 respectively).^[^ ^9^ ^]^ In addition, according to the clinical characteristics of the patients, it was discovered that individuals with low expression of circCK1γ3 had a significantly lower overall survival rate compared to those with high expression. The Cox regression analysis confirmed low expression of circCK1γ3 has an independent risk factor for CRC patients with an HR value of 2.539, and the high expression of circWDR37 is indicated by an HR value of 5.651 (Figure 2g). Overall, these results suggest that the decreased expression of circCK1γ3 and the increased expression of circWDR37 in plasma are associated with the occurrence and the poor prognosis of CRC patients. This indicates that both of these cirRNAs may serve as potential biomarkers for CRC screening and diagnosis.

### 2.2 Developing Multiplexed RPA for circRNA Detection

**Figure 3:**
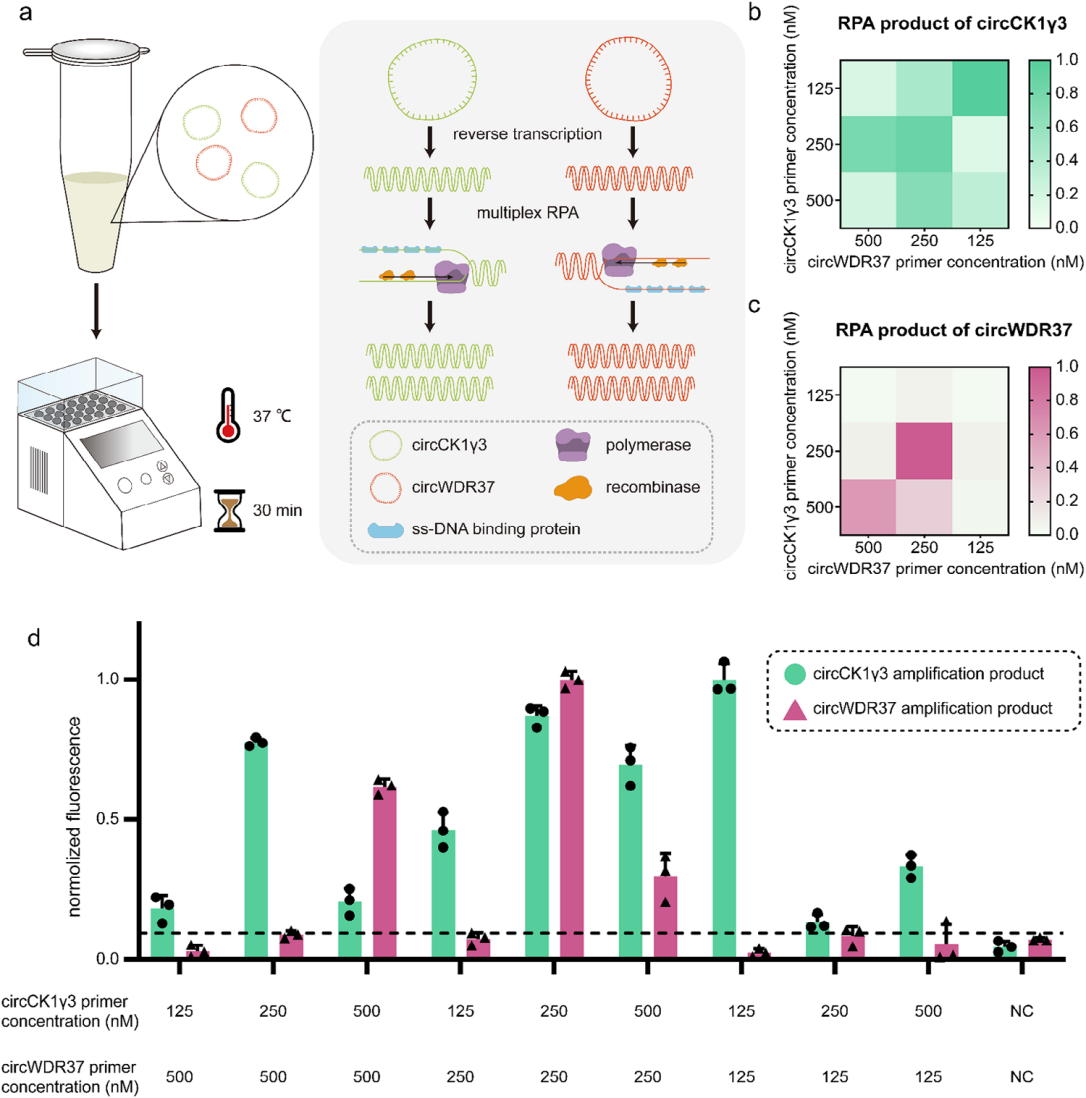
Multiplexed RPA detection system. a) Schematic illustration of multiplex RPA detection of two circRNAs. b,c) Heatmap of primer optimization. Each cell represented a primer combination. d) Multiplex RPA amplification effect under different primer concentration combinations.

Given that the concurrent detection of circCK1γ3 and circWDR37 significantly enhances the precision of colorectal cancer (CRC) screening, we aim to establish a platform for dual circRNA detection. The first step was to establish a multiplex RPA system (**Figure** **3**a). To achieve the optimal amplification efficiency, we designed 9 pairs of RPA primers for circCK1γ3 and evaluated their performance (Figure 3b). The results showed that F2R3 had the highest amplification efficiency. The primers for circWDR37 amplification were also optimized (Figure 3c), thus we attempted to conduct simultaneous amplification of two circRNAs in a single reaction system.

We found that simply combining the two systems would not lead to the successful amplification of both circRNAs. This was because one circRNA exerted a dominant effect on the reaction, thereby inhibiting the amplification of the other circRNA. To achieve multiple RPA, it is essential to maintain synchronized and efficient amplification efficiency for both circRNAs. As a result, we adjusted the concentration of primers in the reaction system, resulting in a notable amplification of both circRNAs at a final primer concentration of 250 nM (Figure 3d). In addition, we calculated the ratios of circCK1γ3 and circWDR37 products under different conditions. It was found that the product ratio was closest to 1 when the primer concentration of both circRNAs was 250 nM. This suggested that this primer concentration can enable efficient and balanced amplification of circCK1γ3 and circWDR37. In summary, we have completed the initial phase of establishing the dual circRNA detection platform, specifically the development of the multiplex RPA amplification system.

### 2.3 Optimizing Multiplexed CRISPR‐Based Detection for circRNA Identification

It has been demonstrated that Cas13a from *Leptotrichia wadei* (LwaCas13a) and Cas13b from *Prevotella sp. MA2016* (PsmCas13b) can be used orthogonally to detect two indicators in the same reaction system (Cas13a favors U‐rich sequences, Cas13b favors A‐rich sequences).^[^ ^12^ ^]^ Moreover, both enzymes exhibit optimal reactivity at 37 °C and can be coupled with RPA, thereby establishing a theoretical foundation for dual circRNA detection. To extend the assay to two circRNAs, we synthesized and purified the LwaCas13a and PsmCas13b protein (Figure S2, Supporting Information). We also designed three crRNAs targeting circCK1γ3, and prepared a FAM‐labeled poly A probe to match the cleavage preferences of the PsmCas13b protein.

**Figure 4:**
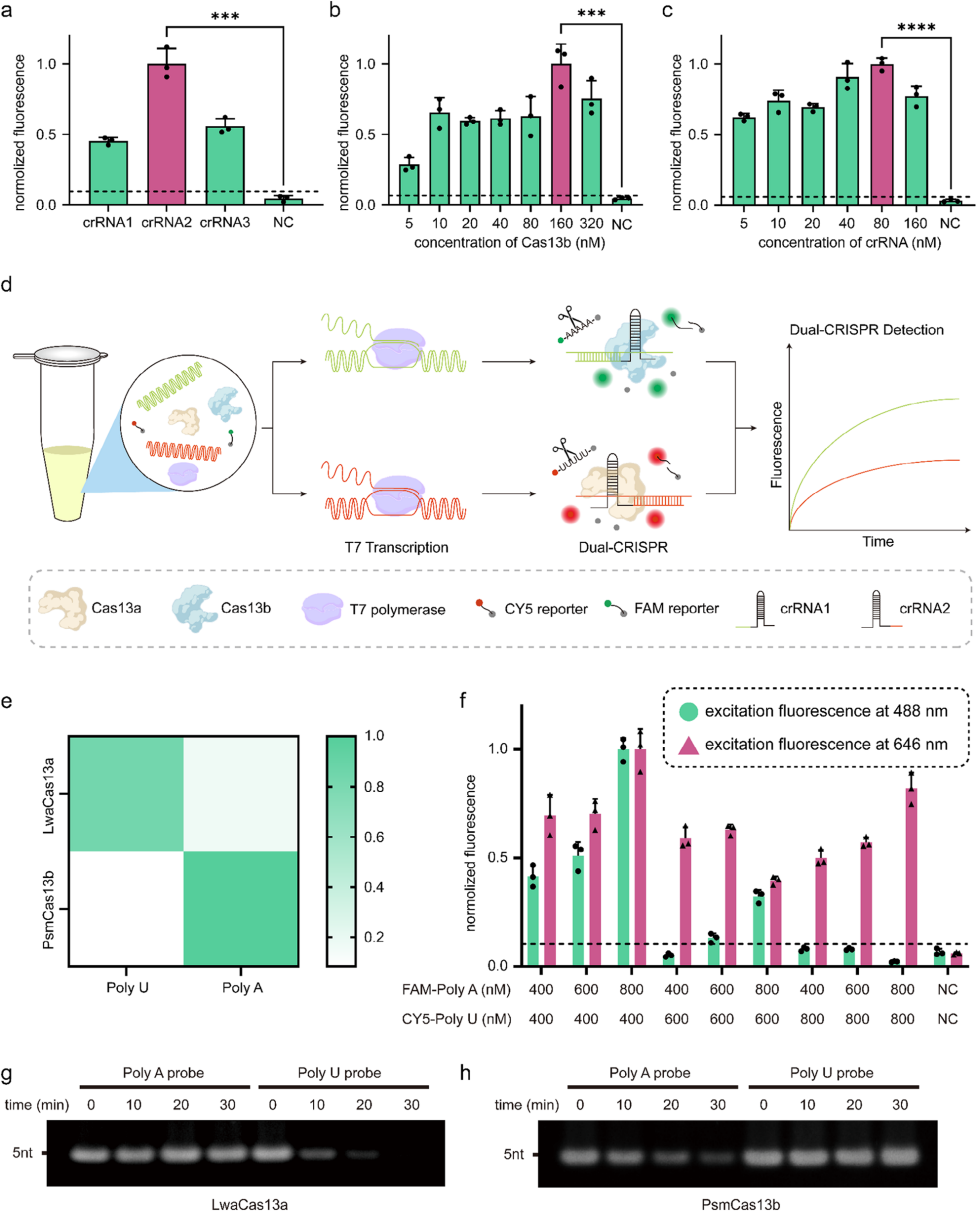
Optimizing of multiplexed CRISPR‐based detection method. a–c) Bar plot of PsmCas13b reaction system optimization. Error bar represents the mean and standard deviation. d) Schematic illustration of multiplexed CRISPR‐based detection for circRNAs. e) Heatmap of LwaCas13a and PsmCas13b orthogonal detection. f) Bar plot of FAM‐Poly A probe and CY5‐Poly U probe concentration optimization. g,h), Electropherograms of LwaCas13a and PsmCas13b cutting probes. Times represent CRISPR reaction times.

We first optimized the reaction system of PsmCas13b for circCK1γ3. The results showed that the highest efficiency of detection was achieved when the concentration of crRNA2 was 80 nM and the concentration of Cas13b protein was 160 nM (**Figure** **4**a–c). Moreover, we demonstrated that both proteins can function orthogonally in the same system, because they tend to cleave poly A probe and poly U probe, respectively (Figure 4e). The specificity of the two Cas13 proteins for probe cleavage was then verified using PAGE electrophoresis. As illustrated in Figure 4g,h, the activated LwaCas13a protein demonstrates significant cleavage of the poly‐U probe within 30 min. In contrast, the PsmCas13b protein does not exhibit this behavior.

**Figure 5:**
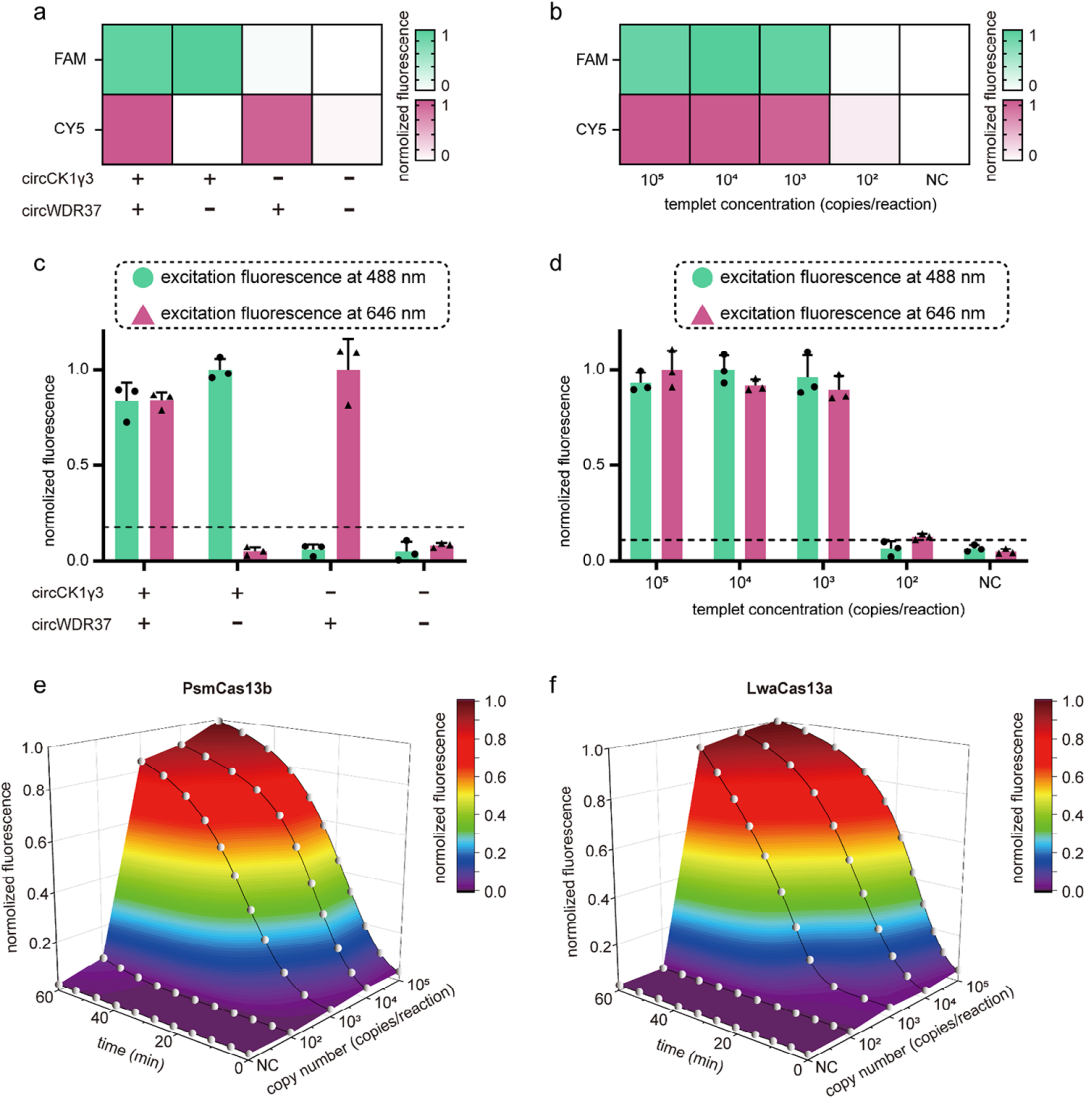
Detection performance of multiplexed CRISPR‐based detection method. a) Heat map of specificity of multiplexed CRISPR‐based detection method. b) Heat map of sensitivity of multiplexed CRISPR‐based detection method. c) Bar plot of specificity of multiplexed CRISPR‐based detection method. d) Bar plot of sensitivity of multiplexed CRISPR‐based detection method. e,f) 3D plot of PsmCas13b and LwaCas13a fluorescence curves of samples with different concentrations.

Leveraging this approach, we utilized LwaCas13a to cleave the CY5‐labeled poly‐U probe for the detection of circWDR37 and PsmCas13b to cleave the FAM‐labeled poly‐A probe for the detection of circCK1γ3 (Figure 4d). To simplify operations and minimize the risk of aerosol contamination during pipetting, we further integrated dual RPA amplification and dual CRISPR‐based detection into a single reaction system, thereby developing a one‐pot detection method. It was observed that the concentration of the probe significantly influenced the intensity of the final fluorescence signal. As illustrated in Figure 4f, an excessively high concentration of the CY5‐labeled probe was observed to diminish the overall fluorescence intensity. Ultimately, the optimal concentrations of the FAM‐labeled and CY5‐labeled probes were determined to be 800 and 400 nM, respectively. Under these conditions, both fluorescence signals achieved their highest intensities, and the detection of the two indicators was well‐balanced. We subsequently evaluated the sensitivity and specificity of the one‐pot method for detecting circCK1γ3 and circWDR37. As shown in **Figure** **5**a–f, the method can detect both circRNAs simultaneously without cross‐reactivity. The detection sensitivity of both circRNAs was 10^3^ copies/reactions.

### 2.4 Establishing and Optimizing of Casµchip Assay

**Figure 6:**
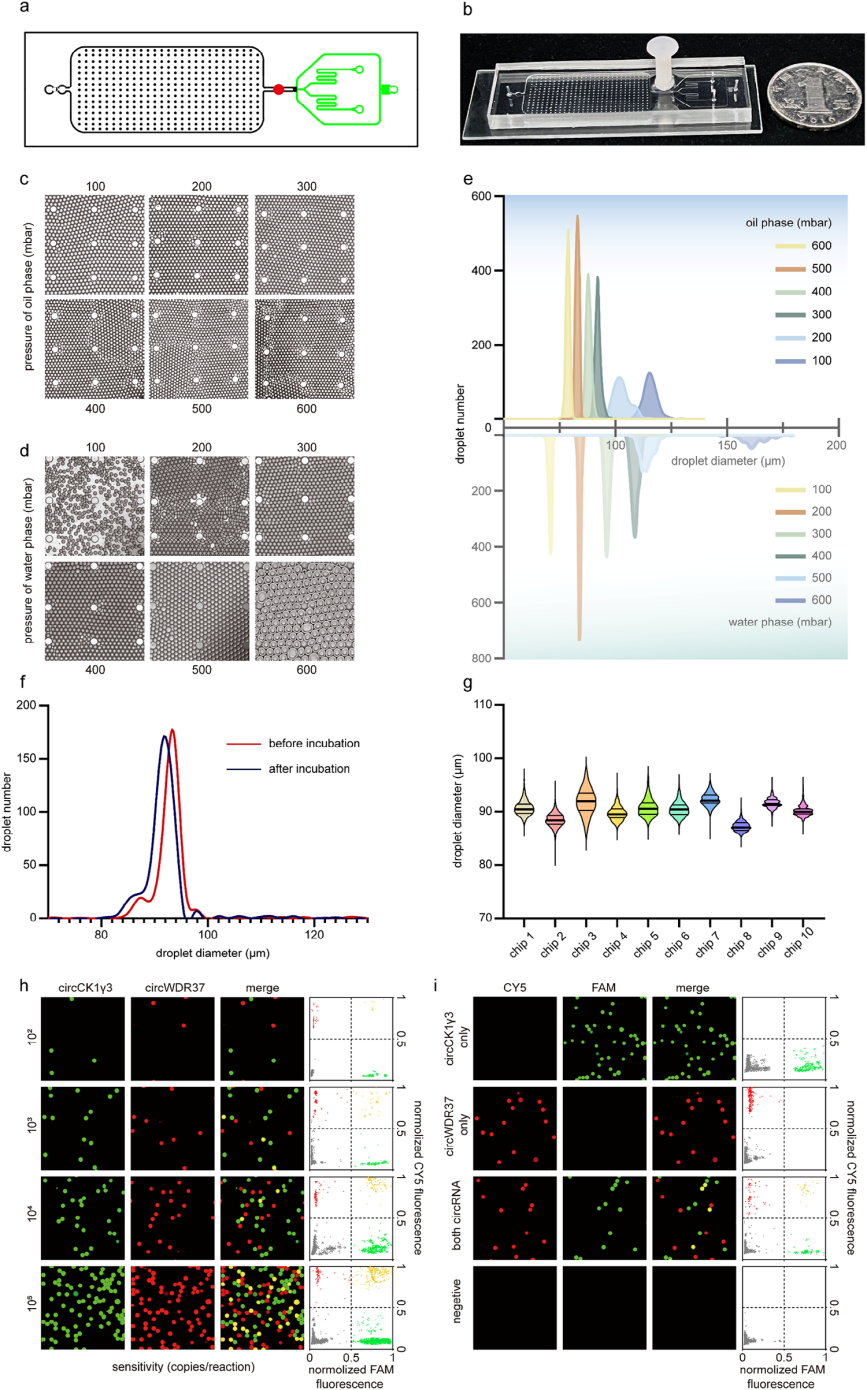
Optimization and detection performance of Casµchip. a,b) The schematic diagram of the chip structure and photographic images of the chip. c,d) Physical diagrams of droplet generation at different oil and liquid phase pressures. e) Statistical plots of droplet size generated at different oil and liquid phase pressures. f) Line graph of droplet size distribution. Incubation conditions were 37 °C for 30 min. g) Violin plot of droplet size distribution in each chip. h) Detection sensitivity of Casµchip, including physical plots under FAM and CY5 channels, fluorescence merge results, and statistical distributions of all droplets. Each row represents a different template concentration. i) Detection specificity of Casµchip.

To achieve quantitative detection of tumor markers and further enhance detection sensitivity, we developed a CRISPR‐based dual‐circRNA detection microfluidic chip, designated as CasµChip. The schematic diagram of the chip structure and photographic images of the chip are shown in **Figure** **6**a,b, respectively. In comparison to previous work,^[^ ^9^ ^]^ Casµchip incorporates both droplet generation chip and droplet detection chip into a single unit, thereby eliminating the necessity for pipetting. The Casµchip employs a water‐in‐oil emulsion process to generate droplets, utilizing structures including an oil input phase, two aqueous input phases, and a droplet‐generating cross zone. Among them, the oil‐phase input is designed with a multi‐layer blocking structure, which prevents impurities from entering the channel. Then, the Casµchip automatically leads the droplets into the droplet detection zone. This region measures 100 µm in height and consists of numerous polydimethylsiloxane (PDMS) pillars for structural support. Ultimately, the excess oil is expelled from the chip via the waste outlet. This process is preceded by a blocking structure that prevents droplets from prematurely exiting the system. Upon completion of the aforementioned steps, the chip can be heated to initiate the RPA‐CRISPR one‐pot reactions. Subsequently, a fluorescence reading device can be utilized to directly observe and enumerate the positive droplets. Overall, the CasµChip requires ≈30 s to cover the entire droplet detection area without the need for manual pipetting, thereby enhancing operational convenience. (Figure S4, Supporting Information). The droplet generation process is documented in Video S1 (Supporting Information). Uniform‐sized droplets are formed in the microfluidic channel and pneumatically propelled to the storage and detection chamber. Following this process, ≈30 000 droplets are systematically positioned within the detection chamber to facilitate subsequent one‐pot reactions and quantitative analysis.

In order to generate stable droplets, the pressures of the aqueous and oil phases were optimized. Initially, the pressure of the aqueous phase was fixed at 300 mbar. Upon gradually increasing the pressure of the oil phase from 100 to 600 mbar, the diameter of the generated droplets was reduced from 115 to 85 µm (Figure 6c). Subsequently, the pressure of the oil phase was fixed at 300 mbar, and when the pressure of the water phase was gradually increased from 100 to 600 mbar, the diameter of the generated droplets was increased from 70 to 160 µm (Figure 6d). As illustrated in Figure 6e, droplet stability was significantly diminished when the diameter exceeded 100 µm. This phenomenon can be attributed to the droplet detection zone having a height of 100 µm, which results in oversize droplets being compressed into irregular shapes. Moreover, droplets that are too small may fail to adequately fill the entire detection zone, potentially compromising the accuracy of the final count. Consequently, the pressure settings were optimized such that both the oil phase and water phase were maintained at 300 mbar, thereby facilitating the generation of stable droplets with an average size of 90 µm. Furthermore, the generated droplets were subjected to a heating process at 37 °C for 30 min, and the size distribution remained unchanged (Figure 6f). The droplet generation process was replicated across ten chips, yielding a consistent size distribution with an average diameter of ≈90 µm and no significant variations (Figure 6g). It is worth mentioning that the chip demonstrates good stability. We examined the concentration of circWDR37 and circCK1γ3 in the same sample using 8 different microchips, 4 of which were freshly prepared and 4 of which had been prepared six months ago. The results showed good reproducibility for all microchips. (CV = 1.3% and 4.1%, respectively) (Figure S3, Supporting Information).

### 2.5 Clinical Application Analysis of CasµChip

**Figure 7:**
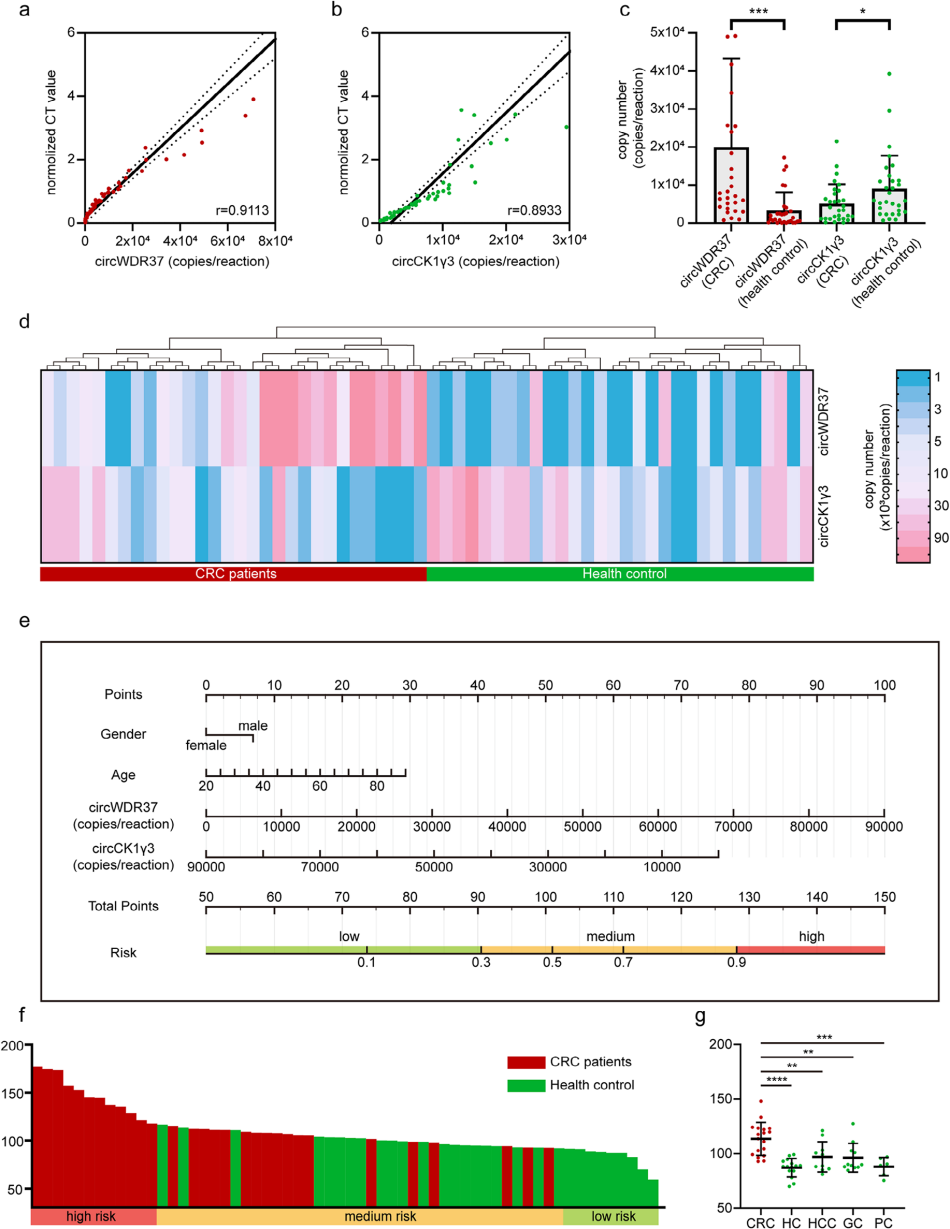
Effectiveness of Casµchip for screening CRC clinical samples. a,b) Correlation of Casµchip quantification of circWDR37 and circCK1γ3 in clinical samples with qPCR results (*n* = 60). c) Statistical plots of circWDR37 and circCK1γ3 levels in clinical samples using Casµchip. The statistical method was unpaired t‐test. d) Clustering heatmap of circWDR37 and circCK1γ3 expression in clinical samples detected by Casµchip (*n* = 60). e) Nomogram of patients gender, age, circWDR37 and circCK1γ3 expression combination. The corresponding CRC risk values were obtained by summing the scores of all factors. f) The 60 patients were ranked according to risk factors, with high risk on the left and low risk on the right. g) The results of clinical samples by blind testing. Vertical coordinates are risk points. CRC represented colorectal cancer (*n* = 18). HC represented health control (*n* = 15). HCC represented hepatocellular carcinoma (*n* = 10). GC represented gastric cancer (*n* = 11). PC represented pancreatic cancer (*n* = 6).

We detected circWDR37 and circCK1γ3 levels in the plasma of 60 clinical cases by Casµchip and qPCR simultaneously, and the results showed that the correlation between the two methods was 0.9113 (circWDR37) and 0.8933 (circCK1γ3), respectively (**Figure** **7**a,b). Furthermore, the results of Casµchip demonstrated that circWDR37 levels were significantly elevated in the CRC group (*P* = 0.0003), while circDK1γ3 levels were significantly decreased (*P* = 0.0359) (Figure 7c). As a whole, the CRC group clustered the majority of samples with high circWDR37 expression and low circCK1γ3 expression (Figure 7d). To enhance the integration of these two biomarkers in the context of CRC screening, a logistic regression analysis was employed to quantify the impact of two biomarkers on CRC screening. Additionally, epidemiological study found that male sex and increasing age have consistently shown strong associations with CRC incidence.^[^ ^13^ ^]^ As a result, age and gender were incorporated into the model to assign each screened patient a risk point, with patients below 90 designated as low CRC risk and those above 128 classified as high CRC risk (Figure 7e). This risk scoring system was employed to evaluate a cohort comprising 30 patients diagnosed with CRC and 30 healthy controls. The results demonstrated that all individuals classified in the high‐risk category were CRC patients, while all individuals in the low‐risk category were healthy controls (Figure 7f). Furthermore, a blinded method was employed in the testing of 60 samples, which included patients with colorectal cancer (CRC), healthy controls (HC), hepatocellular carcinoma (HCC), gastric cancer (GC), and pancreatic cancer (PC). The results showed that the risk points of colorectal cancer patients were significantly higher than that of other groups, which indicated the high specificity of the method (Figure 7g).

## 3 Discussion

The development of non‐invasive, highly accurate screening tools for CRC remains a critical unmet need in clinical oncology.^[^ ^14^ ^]^ While colonoscopy remains the gold standard for CRC detection, its reliance on patient compliance and resource‐intensive nature limits its scalability for population‐wide screening.^[^ ^15^ ^]^ Existing alternatives, such as fecal immunochemical tests (FITs) or blood‐based biomarkers like CEA and CA199, suffer from suboptimal sensitivity or specificity. These limitations underscore the urgent demand for novel biomarkers and detection platforms that combine high diagnostic performance with practicality for large‐scale implementation.^[^ ^16^ ^]^ In this context, our study introduces a dual‐circRNA‐based detection system, Casµchip, which addresses both biological and technical challenges in CRC screening.

Unlike conventional protein‐based biomarkers, circRNAs exhibit tissue and disease‐specific expression patterns, with enhanced stability in peripheral blood due to their covalently closed circular structure.^[^ ^17^ ^]^ Numerous studies have indicated an association between circRNAs and critical aspects of CRC, including progression and metastasis. For example, circFBXW4 competitively binds to miR‐338‐5p and prevents it from interacting with and repressing its target SLC5A7, thus suppressing the progression of CRC.^[^ ^18^ ^]^ CircHIPK2‐EIF4A3 axis contributes to cell growth in intestinal epithelial of colitis and CRC by enhancing TAZ translation.^[^ ^19^ ^]^ CircNOLC1 interacting with AZGP1 and circNOLC1/miR‐212‐5p/c‐Met axis plays a key role in oxidative pentose phosphate pathway‐mediated colorectal cancer liver metastasis.^[^ ^20^ ^]^ In this study, we identified circWDR37 and circCK1γ3 as combined biomarkers for CRC screening. The improvement in AUC from 0.754 (µDCR) to 0.808 (Casµchip) highlights how multiplexed detection synergistically enhance diagnostic power. This aligns with emerging evidence that multi‐analyte panels better capture the molecular heterogeneity of CRC, particularly at early stages when tumor‐derived signals in circulation may be scarce.^[^ ^21^ ^]^ Importantly, the differential expression patterns of these circRNAs across CRC progression stages suggest their potential utility not only in detection but also in monitoring therapeutic response or recurrence.

Technologically, the Casµchip platform overcomes two critical bottlenecks in circRNA diagnostics: quantification and orthogonality. By integrating droplet microfluidics with dual RPA‐CRISPR systems, we achieved quantification of two circRNAs without cross‐reactivity, while eliminating transfer steps that previously compromised workflow efficiency and introduced contamination risks. The readout via FAM and CY5 channels provides an interface suitable for low‐resource settings, which contrasts favorably with PCR‐based methods requiring specialized equipment.^[^ ^22^ ^]^ It is noteworthy that Casµchip demonstrates exceptional stability and reproducibility, with a shelf life of at least six months when stored at room temperature. Furthermore, the cost of Casµchip is economically viable. Upon detailed calculation, it has been determined that the microarray costs $1.5, the reagents cost $2.5, and the total cost per sample for Casµchip is $4.

However, this study has several limitations that need to be further clarified. The newly identified CRC biomarker circCK1γ3 lacks experimental validation through both cellular models and animal studies, and the precise molecular mechanisms underlying its regulatory effects on CRC progression remain to be fully elucidated. While our droplet system reduces reaction time, the requirement for microfluidic chips and CRISPR‐associated reagents may initially limit its application situations.^[^ ^11^ ^]^ Future iterations could explore lyophilized reagents or smartphone‐based readout systems to enhance point‐of‐care applicability. Luckily, the Casµchip platform's adaptability allows rapid incorporation of additional circRNA biomarkers as they are discovered, creating a scalable framework for precision screening.

## 4 Conclusion

In conclusion, this study addresses a critical gap in the intersection of circRNA biology and clinical diagnostics. By integrating novel biomarker discovery with cutting‐edge engineering, LwaCas13a and PsmCas13b were integrated to achieve specific differentiation and detection of dual targets (circCK1kγ3 and circWDR37) by leveraging their distinct cleavage preferences (Cas13a favors U‐rich sequences, Cas13b favors A‐rich sequences). This method effectively circumvents the cross‐interference issues commonly encountered in multi‐target detection using traditional single‐Cas systems.

## 5 Experimental Section

### Materials

TB Green Premix Ex Taq for qPCR and PrimeScript RT reagent Kit with gDNA Eraser for reverse transcription were purchased from TaKaRa (Japan). LS Total RNA Extraction Reagent (TRIease) were purchased from YEASEN Biotechnology (Shanghai, China). SPINeasy RNA Kit for Tissue (with Lysing Matrix) was obtained from MP Biomedical (USA). Ethanol absolute, trichloromethane and iso‐propyl alcohol were purchased from Linfeng Chemical Reagent Company (Shanghai, China). RPA reagent was purchased from Leshang bio (Wuxi, China) and CRISPR‐Cas13 was got from MAGIGEN bio (Guangzhou, China). Si master was purchased from Zhongxinqiheng (Suzhou, China). SU‐8 photoresist and developer were obtained from Microchem (USA). Sequence of primers and crRNAs used in this work were shown in Table S1 (Supporting Information).

### Patients and Specimens

This study obtained approval from the patients and adhered to the informed consent protocols and ethical standards of Renji Hospital, Shanghai Jiaotong University School of Medicine, Shanghai, China. The colorectal cancer (CRC) tissues were procured from surgically resected specimens, while the corresponding adjacent non‐cancerous tissues were obtained from sites ≈5–10 cm away from the tumor margin. A total of 60 pairs of CRC tissues were included, and tumor staging was conducted in accordance with the sixth edition of TNM classification of the International Union Against Cancer (UICC, 2009).

Concurrently with the tumor tissues, peripheral blood samples were collected. In total, 300 blood samples were gathered for control purposes, comprising 60 from healthy donors, 60 from patients with benign conditions such as colon polyps, ulcerative colitis, viral colitis, colorectal adenoma, prostate hyperplasia, and gastritis, 60 from cancer patients with gastric cancer, liver cancer, esophageal cancer, pancreatic cancer, prostate cancer, and peritoneal tumors, and 120 from CRC patients in different stage. All samples used in this study followed the informed consent and agreement of patients as well as the ethical regulations of Shanghai Jiaotong University School of Medicine Renji Hospital. Ethics (RA‐2023‐629).

### RNA Extraction

Tissue samples were thoroughly homogenized using the FastPrep instrument (MP Biomedicals, USA). Following this, the samples were centrifuged at 14 000 g for 10 min, and the resulting supernatant was collected. Initially, samples were washed with an equal volume of absolute ethanol and Wash Buffer R. DNaseI was then introduced to the samples and allowed to incubate for 15 min to eliminate residual DNA. Subsequently, a further washing step was carried out using Wash Buffer R, and the RNA was eluted with nuclease‐free water to yield tissue RNA.

As for liquid samples, 0.75 mL of LS Total RNA Extraction Reagent and 0.2 mL of chloroform were added to 0.25 mL of the sample. Following centrifugation at 12 000 g for 15 min at 4 °C, the upper layer was retained, and an equivalent volume of isopropanol was added. After undergoing alcohol washing, the total RNA within the liquid sample was obtained.

### Reverse Transcription and Real Time Quantitative PCR

In accordance with the manufacturer's instructions, RNA was reverse transcribed into cDNA. The PCR reaction was carried out with the TB Green Premix Ex Taq (TaKaRa, Japan) using the applied biosystems 7500 real time PCR system. The PCR analysis conditions were as follows: initial denaturation for 30 s at 95 °C, followed by 40 cycles of denaturation at 95 °C for 5 s and annealing at 60 °C for 34 s.

### Recombinase Polymerase Amplification

Recombinase polymerase amplification (RPA) reactions were performed using a kit (Leshang bio, Wuxi, China) according to the manufacturer's protocol. Specifically, the reactions were carried out in a total volume of 50 µL, which included RPA enzymes, 2 µL RNA, 25 µL buffer, 16 µL water, 2 µL of each primer, and 3 µL initiation reagent. Subsequently, all reactions were incubation at 39 °C for 20 min.

### Expression and Purification of LwaCas13a and PsmCas13b Proteins

The target gene encoding LwaCas13a and PsmCas13b was cloned into the expression vector with a His‐tag. The recombinant plasmid was transformed into E. coli. A single colony was inoculated into medium containing 50 µg mL^−1^ kanamycin and cultured overnight at 37 °C. The pre‐culture was diluted 1:100 into fresh medium and grown at 37 °C until the optical density at 600 nm reached 0.6. Protein expression was induced by adding 0.5 mM IPTG, followed by incubation at 18 °C for 16 h. Cells were harvested by centrifugation at 6000 g for 15 min at 4 °C.

For purification, the cell pellet was resuspended in 20 mM Tris‐HCl and 300 mM NaCl. The lysate was centrifuged at 12 000 g for 30 min at 4 °C to remove debris. The supernatant was loaded onto a Ni‐NTA affinity column equilibrated with lysis buffer. After washing, the target protein was eluted. The eluted protein was further purified by size‐exclusion chromatography. Protein purity and molecular weight were confirmed by SDS‐PAGE.

### CRISPR/Cas13b Detection System

The primary components of the CRISPR/Cas13b detection system include the Cas13b protein, crRNA, single‐stranded RNA fluorescent probe, RNase inhibitor, and buffer from MAGIGEN bio, Guangzhou, China. Reactions were carried out in a total volume of 50 µL and incubated at 37 °C for 20 to 40 min. The outcomes were assessed using a fluorescence reader or visually, employing an excitation light wavelength of 488 nm (FAM) or 646 nm (CY5) and an emission light wavelength of 510 nm (FAM) or 670 nm (CY5).

### Casµchip Design and Fabrication

The Casµchip was initially designed in AutoCAD 2022 and subsequently constructed. As illustrated in Figure 6a, the chip consists primarily of two key components: the droplet generation region (highlighted in green) and the droplet storage and detection region (depicted in black). Microcolumns, measuring 100 microns in height, are positioned within the droplet detection region to reinforce the structural integrity and mitigate collapse. Furthermore, a screw valve (indicated in red) is integrated between the droplet generation and detection regions to facilitate fluid regulation, ensuring proper droplet reaction within the designated detection area.

To create the cruciform droplet generation zone, SU8 photoresist was deposited at two distinct heights. A spin‐coating technique was used to create a layer of ≈70 µm in thickness using SU‐8 2075 at 1800 rpm for 30 s. Similarly, a 35 µm layer was produced using SU‐8 3050 at 4000 rpm for 30 s on the MA6 system manufactured by Karl Suss Corp. in Germany. A molding technique was employed for the fabrication of the PDMS chip. The PDMS precursor mixture was prepared at a weight ratio of 10:1 (base to curing agent) and carefully poured onto the master. After vacuum treatment to remove bubbles, the mixture was cured at 90 °C for 2 h. The resulting cured PDMS replica was then delicately separated from the master and securely bonded to a glass slide using plasma to ensure leak‐proof integration.

### Digital Dual CRISPR/Cas13a‐RPA System

The reaction system was partitioned into two segments and placed in tubes A and B, respectively. Tube A contained RPA lyophilized powder, 5 µL NTP, 2 µL FAM probe, 2 µL CY5 probe, 4 µL template, 4 µL primer, and 20 µL buffer. Tube B comprised 1 µL Cas13a, 3 µL Cas13b, 0.5 µL RNase inhibitor, 0.5 µL T7 transcriptase, 2 µL crRNA, 3 µL magnesium acetate, and 30 µL buffer. Under pressure, reagent A and reagent B were introduced into the liquid phase channel through injection ports of the chip, respectively. Concurrently, mineral oil was introduced into the oil tunnel through another injection port. This process led to the generation of roughly thousands of water‐in‐oil droplets, measuring ≈90 µm in size, in the designated droplet generation. Incubation at 37 °C for 15 min enabled the simultaneous occurrence of dual‐RPA and CRISPR reactions within the droplets.

### Digital Quantification

Based on the storage capacity of the chip, up to 30 000 droplets were generated and subsequently analyzed in each experiment. The target concentration was determined using the Poisson distribution. The fluorescence intensity of the droplets was assessed by the Nebula auto digital PCR system (ThunderBio Life Sciences), a specialized chip‐reading device.

### Nomogram Results

The primary outcome variable was defined as dead, with 0 representing death and 1 representing survival. The predictor variables employed in this study included gender, age, circWDR37, and circCK1γ3.Given that gender is a categorical variable, 0 and 1 were used to represent males and females respectively, and were defined as int variables. The nomogram results were output through the implementation of multifactorial logistic regression analysis.

### Statistical Analyses

The analysis of real‐time PCR results in all experiments employed the 2‐ΔΔCt method. Differences in circRNA levels were assessed using an unpaired t‐test. For the generation of Receiver Operating Characteristic (ROC) curves, SPSS Statistics 27 and GraphPad Prism 8.0.2 were utilized. Kaplan‐Meier curves were employed to evaluate the probability of differences in overall survival (OS), with the significance of differences assessed using a log‐rank test. The significance level for all analyses was set at a *P*‐value of 0.05. *P*‐values less than 0.05, 0.01, 0.001, and 0.0001 were denoted by 1, 2, 3, and 4 asterisks, respectively.

## Conflict of Interest

The authors declare no conflict of interest.

## Author Contributions

J.X., Y.L. and J.Z. contributed equally to this work and should be considered co‐first authors. J.X. and Y.L. designed and performed the experiments, analyzed the data, and wrote the manuscript. J.Z., S.Y., and T.L. designed and performed the experiments. H.H. were responsible for clinical sample collection. Q.L., H.W., and L.C. assisted in the analysis of data and edited the language of the manuscript. Z.A., M.L., and H.W. designed and supervised the research and wrote the manuscript. All authors read, revised, and approved the final manuscript.

## Supporting information



Supporting Information

Supplemental Video 1

## Data Availability

The data that support the findings of this study are available in the supplementary material of this article.

## References

[1] Y. Guo , Y. Guo , C. Chen , D. Fan , X. Wu , L. Zhao , B. Shao , Z. Sun , Z. Ji , Mol. Cancer 2021, 20, 93. 34172072 10.1186/s12943-021-01372-0 PMC8229759

[2] U. Ladabaum , J. A. Dominitz , C. Kahi , R. E. Schoen , Gastroenterology 2020, 158, 418. 31394083 10.1053/j.gastro.2019.06.043

[3] A. N. Burnett‐Hartman , J. K. Lee , J. Demb , S. Gupta , Gastroenterology 2021, 160, 1041. 33417940 10.1053/j.gastro.2020.12.068 PMC8273929

[4] L. Kelly , Nurs. Stand 2022, 37, 77. 10.7748/ns.2022.e11901 35257537

[5] D. K. Rex , R. C. Boland , J. A. Dominitz , F. M. Giardiello , D. A. Johnson , T. Kaltenbach , T. R. Levin , D. Lieberman , D. J. Robertson , Am. J. Gastroenterol. 2017, 112, 1016. 28555630 10.1038/ajg.2017.174

[6] a) Q. Liu , J. Chen , Y. Wang , S. Li , C. Jia , J. Song , F. Li , Brief Bioinform 2021, 22, bbaa124; 32608476 10.1093/bib/bbaa124 PMC8599298

[7] F. Di Cesare , A. Vignoli , C. Luchinat , L. Tenori , E. Saccenti , Metabolites 2023, 13, 296. 36837915 10.3390/metabo13020296 PMC9965766

[8] S. S. Choi , S. E. Kim , S. Y. Oh , Y. H. Ahn , Biomedicines 2022, 10, 871.35453621

[9] J. Xu , L. Cao , S. Yang , Y. Jian , Y. Liu , Z. Shen , Q. Liu , X. Chen , M. Li , S. Li , X. Zuo , M. Li , H. Wang , Aggregate 2024, 6, 663.

[10] C. Xue , G. Li , Q. Zheng , X. Gu , Z. Bao , J. Lu , L. Li , Molecular Cancer 2022, 21, 108. 35513849 10.1186/s12943-022-01582-0 PMC9074313

[11] M. Patchsung , A. Homchan , K. Aphicho , S. Suraritdechachai , T. Wanitchanon , A. Pattama , K. Sappakhaw , P. Meesawat , T. Wongsatit , A. Athipanyasilp , K. Jantarug , N. Athipanyasilp , J. Buahom , S. Visanpattanasin , N. Niljianskul , P. Chaiyen , R. Tinikul , N. Wichukchinda , S. Mahasirimongkol , R. Sirijatuphat , N. Angkasekwinai , M. A. Crone , P. S. Freemont , J. Joung , A. Ladha , O. Abudayyeh , J. Gootenberg , F. Zhang , C. Chewapreecha , S. Chanarat , et al., The CRISPR Journal 2023, 6, 99. 36367987 10.1089/crispr.2022.0048 PMC7614457

[12] J. S. Gootenberg , O. O. Abudayyeh , M. J. Kellner , J. Joung , J. J. Collins , F. Zhang , Science 2018, 360, 439. 29449508 10.1126/science.aaq0179 PMC5961727

[13] E. Dekker , P. J. Tanis , J. L. A. Vleugels , P. M. Kasi , M. B. Wallace , Lancet 2019, 394, 1467. 31631858 10.1016/S0140-6736(19)32319-0

[14] H. Brenner , T. Heisser , R. Cardoso , M. Hoffmeister , Nat. Rev. Gastroenterol. Hepatol. 2024, 21, 125. 37794234 10.1038/s41575-023-00847-3

[15] S. G. Patel , J. A. Dominitz , Ann. Intern. Med. 2024, 177, 4. 10.7326/AITC202404160 38588547

[16] J. S. Lin , L. A. Perdue , N. B. Henrikson , S. I. Bean , P. R. Blasi , JAMA, J. Am. Med. Assoc. 2021, 325, 1978. 10.1001/jama.2021.4417 PMC13509038 34003220

[17] W. Y. Zhou , Z. R. Cai , J. Liu , D. S. Wang , H. Q. Ju , R. H. Xu , Mol. Cancer 2020, 19, 172. 33317550 10.1186/s12943-020-01286-3 PMC7734744

[18] W. Song , J. Fu , J. Wu , J. Ren , R. Xiang , C. Kong , T. Fu , Adv. Sci. 2024, 11, 2300129. 10.1002/advs.202300129 PMC11095154 38461489

[19] X. Zeng , J. Tang , Q. Zhang , C. Wang , J. Qi , Y. Wei , J. Xu , K. Yang , Z. Zhou , H. Wu , J. Luo , Y. Jiang , Z. Song , J. Wu , J. Wu , Adv. Sci. 2024, 11, 2401588. 10.1002/advs.202401588 PMC11425914 38981023

[20] M. Yuan , X. Zhang , F. Yue , F. Zhang , S. Jiang , X. Zhou , J. Lv , Z. Zhang , Y. Sun , Z. Chen , H. Wu , X. Liu , X. Yu , B. Wei , K. Jiang , F. Lin , Y. Zuo , S. Ren , Adv. Sci. 2023, 10, 2205229. 10.1002/advs.202205229 PMC10667818 37870214

[21] a) K. Nakamura , G. Hernández , G. G. Sharma , Y. Wada , J. K. Banwait , N. González , J. Perea , F. Balaguer , H. Takamaru , Y. Saito , Y. Toiyama , Y. Kodera , C. R. Boland , L. Bujanda , E. Quintero , A. Goel , Gastroenterology 2022, 163, 1242; 35850198 10.1053/j.gastro.2022.06.089 PMC9613521

[22] Y. Xue , K. Wang , Y. Jiang , Y. Dai , X. Liu , B. Pei , H. Li , H. Xu , G. Zhao , Biosens. Bioelectron. 2024, 247, 115927. 38113694 10.1016/j.bios.2023.115927

