# Supplementary material for: Single‐Droplet Dual‐Target Quantification of circRNA Biomarkers for Colorectal Cancer Screening
Source: Adv Sci (Weinh). 2025 Jun 29;12(38):e06159. doi: 10.1002/advs.202506159 (PMC12520571; doi:10.1002/advs.202506159)
Supplement: Supplementary file 1 — Supporting Information [file ADVS-12-e06159-s002.docx]

Jingsong Xu^1#^, Yu Liu^1#^, Junheng Zhang^1#^, Shuang Yang^1^, Tianming Li^1^, Haiqian Huang^1^, Qian Liu^1^, Hengliang Wang^3^, Li Cao^4✉^, Zhenghua An^3✉^, Min Li^1,2✉^, Hua Wang^1✉^

1 Department of Laboratory Medicine, Renji Hospital, School of Medicine, Shanghai Jiao Tong University, Shanghai, 200127, China

2 Shanghai Jiao Tong University School of Nursing. Shanghai Jiao Tong University, Shanghai, 200025, China

3. State Key Laboratory of Surface Physics, Department of Physics, Fudan University, Shanghai, 200438, China

4. Department of Pathology, The Fifth People’s Hospital of Shanghai, Fudan University, Shanghai, 200137, China

✉**Corresponding authors**

renjijywh@shsmu.edu.cn (H. Wang)

[rjlimin@shsmu.edu.cn](mailto:rjlimin@shsmu.edu.cn) (M. Li)

[anzhenghua@fudan.edu.cn](mailto:anzhenghua@fudan.edu.cn) (ZH. A)

[caoli@5thhospital.com](mailto:caoli@5thhospital.com) (L.Cao)

**Author Contributions**

#These authors contributed equally to this work and should be considered co-first authors


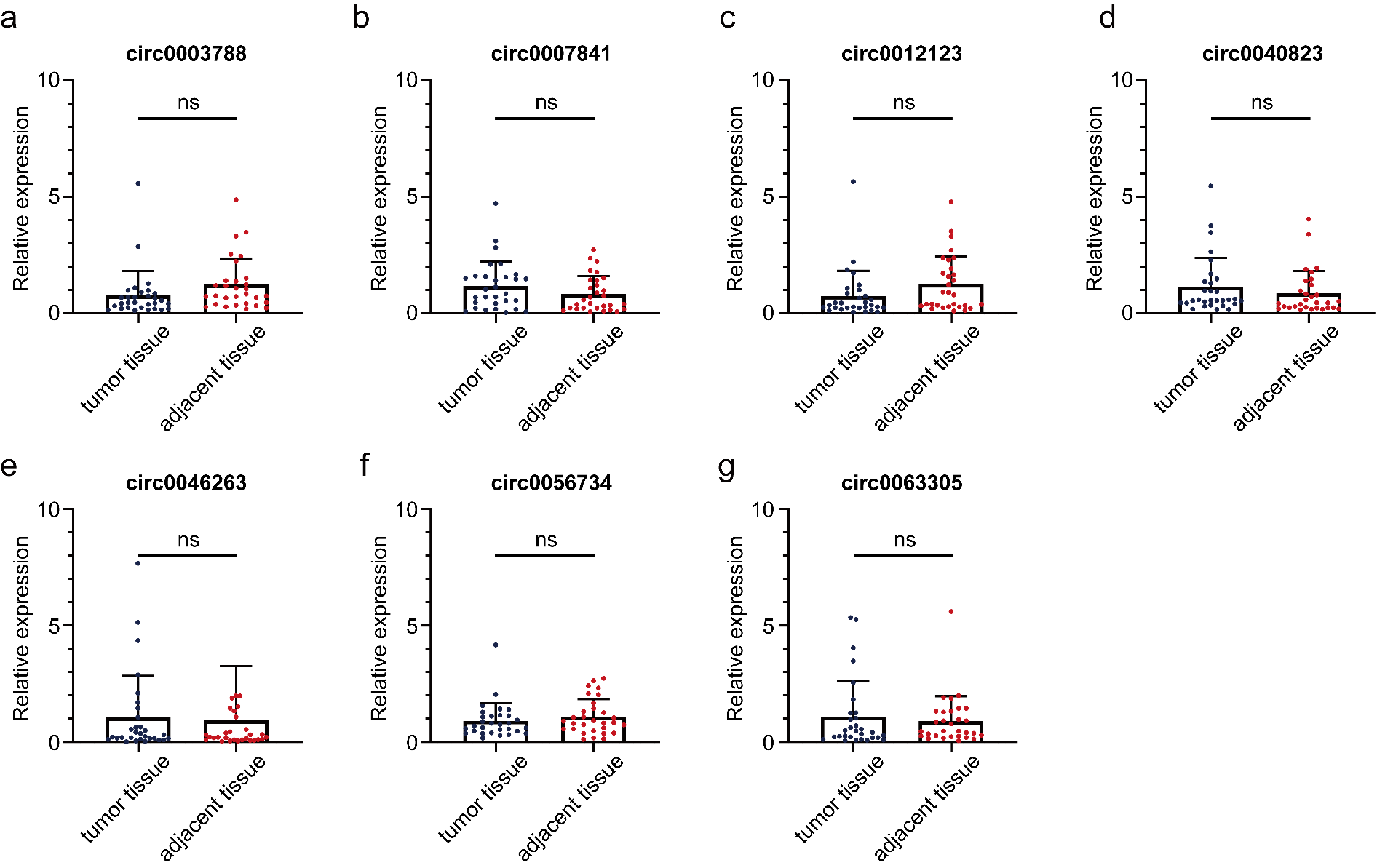


Figure S1. CircRNA expression in tumor tissue and adjacent tissue of colorectal cancer patients. n=64.


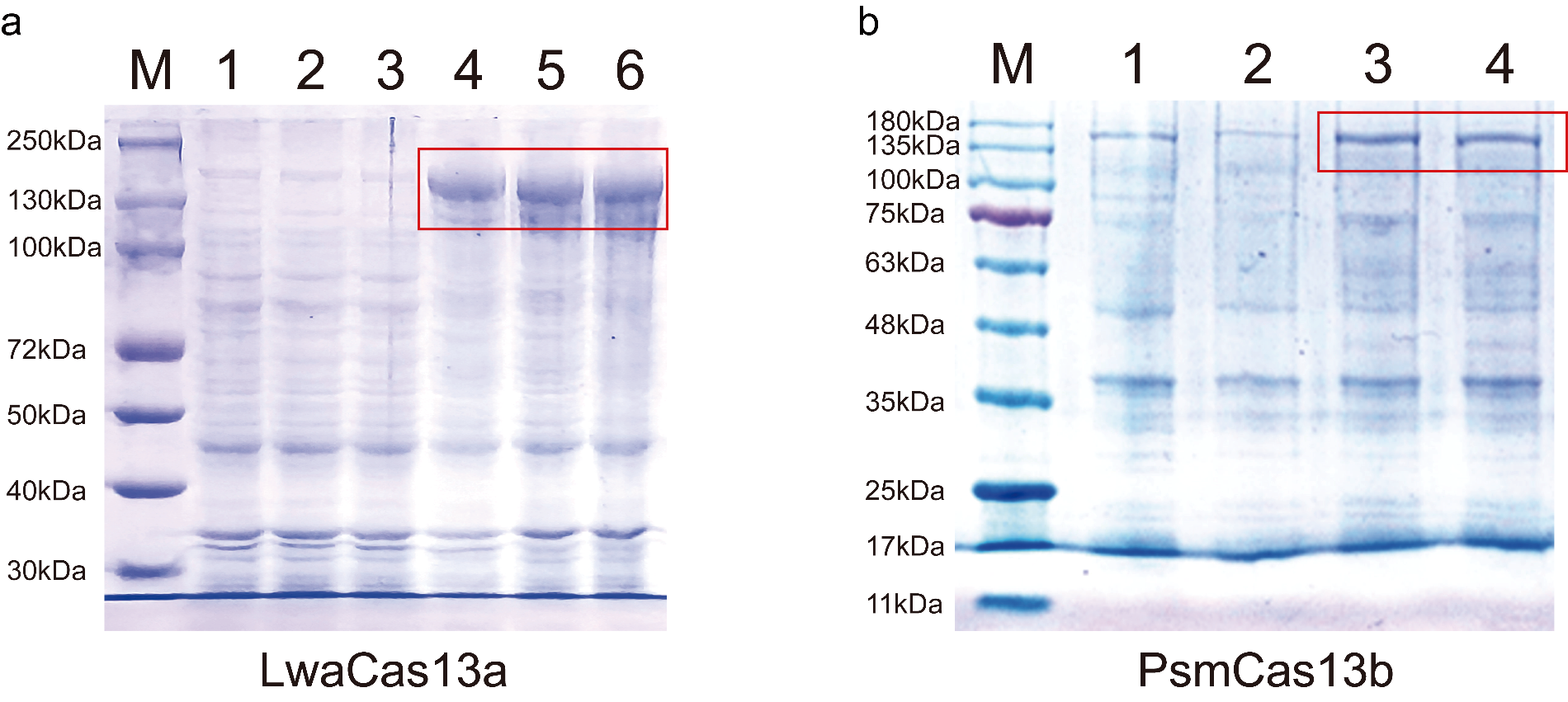


Figure S2. Electropherograms of LwaCas13a and PsmCas13b protein. **a,** Lane 1, 2, 3 are the samples before IPTG-induced expression and lane 4, 5, 6 are the samples after IPTG-induced expression. The red box shows the bands of LwaCas13a. **b,** Lane 1, 2 are the samples before IPTG-induced expression and lane 3, 4 are the samples after IPTG-induced expression. The red box shows the bands of LwaCas13a.


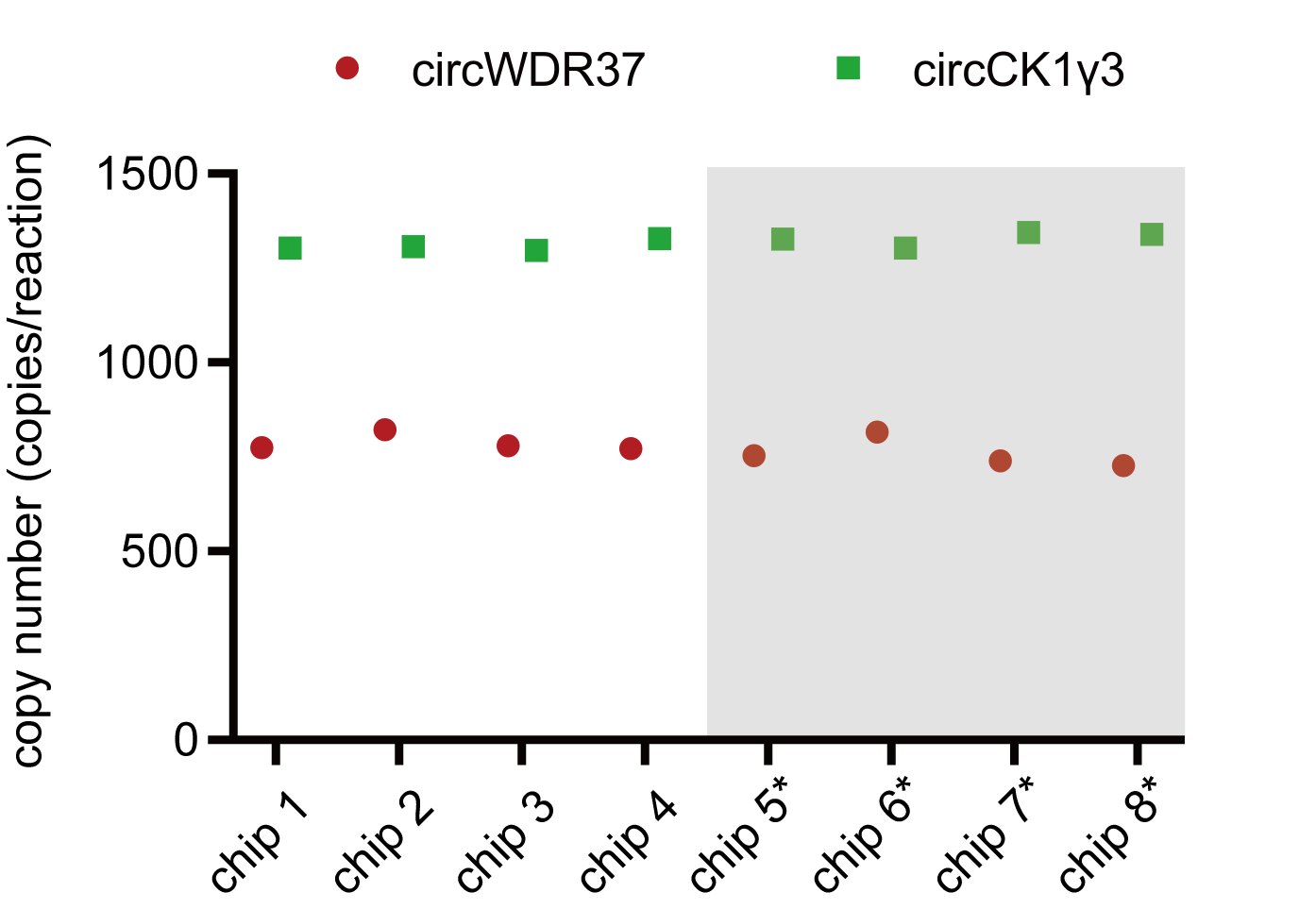


Figure S3. Detection results of the same sample using different chips. Chips 1-4 were freshly prepared, while chips 5-8 had been prepared six months ago and stored at room temperature. Previously prepared chips are identified in the figure with * and a gray background.


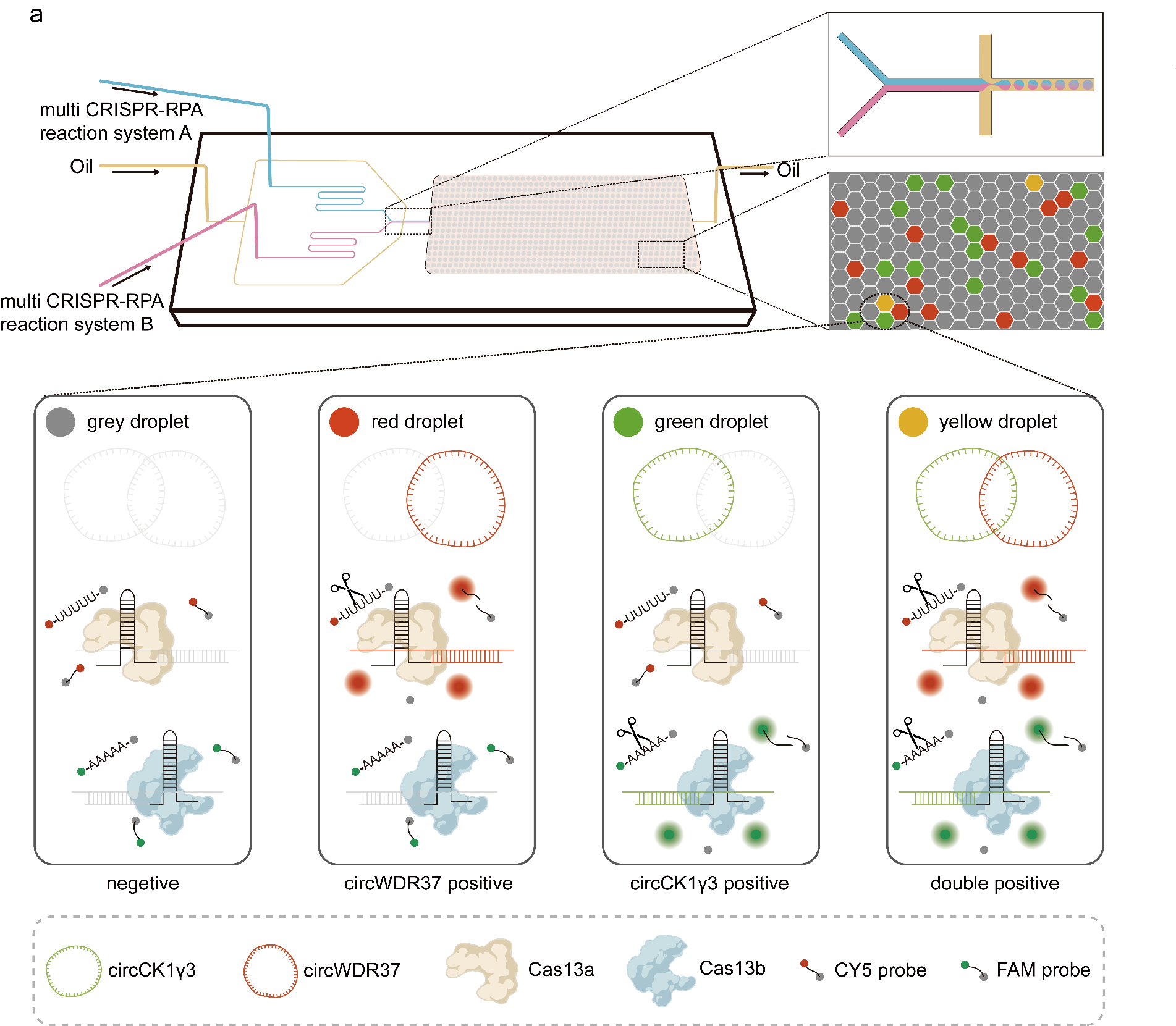


Figure S4. Schematic diagram of casμchip detection. Zoom in to show the inside of the droplet in four states.

**Video S1**

Droplets of uniform size are generated in the droplet generation area and enter the droplet storage and detection area under the action of air pressure. Finally, about 30,000 droplets are laid out in the detection area for subsequent reaction and detection.

| name | sequence (5'-3') |
| --- | --- |
| crRNA1 | AGCAGTTAGGATCTGGAGCTCTCTATCAATGUUGUAGAAGCUUAUCGUUUGGAUAGGUAUGACAAC |
| crRNA2 | GTTAGGATCTGGAGCTCTCTATCAATATCAGUUGUAGAAGCUUAUCGUUUGGAUAGGUAUGACAAC |
| crRNA3 | GGATCTGGAGCTCTCTATCAATATCAGCTCGUUGUAGAAGCUUAUCGUUUGGAUAGGUAUGACAAC |
| poly A probe | FAM-AAAAA-BHQ1 |
| poly U probe | CY5-UUUUU-BHQ2 |
| circCK1γ3-F | CACAAATGAATATGTGGCAATTAAGTTGGAG |
| circCK1γ3-R | TAATACGACTCACTATAGGGACTGTACCAGATGGGTAATGTTAACATTCA |
| circWDR37-F | CATGACAACCGACAAGTGAGACTGTTTGAT |
| circWDR37-R | TAATACGACTGCCTGAAACCACGTTGTCTCCCACGGTGA |

**Table S1 Sequence of primers and crRNAs used in this work.**
